# Supplementary material for: Evidence from oyster suggests an ancient role for Pdx in regulating insulin gene expression in animals
Source: Nat Commun. 2021 May 25;12:3117. doi: 10.1038/s41467-021-23216-7 (PMC8149454; doi:10.1038/s41467-021-23216-7)
Supplement: Supplementary file 1 — Supplementary Information [file 41467_2021_23216_MOESM1_ESM.pdf]

Supplementary information

Evidence from oyster suggests an ancient role for Pdx in regulating insulin gene expression in animals

Fei Xu [1,2 *], Ferdinand Marlétaz [1,3,4], Daria Gavriouchkina [3,5], Xiao Liu [6], Tatjana Sauka-Spengler [5], Guofan Zhang [2,7] and Peter WH Holland [1]

1 Department of Zoology, University of Oxford, Oxford OX1 3SZ, UK
2 Key Laboratory of Experimental Marine Biology, Center for Mega-Science, Institute of Oceanology, Chinese Academy of Sciences, Qingdao 266071, China
3 Molecular Genetics Unit, Okinawa Institute of Science and Technology Graduate University, Okinawa 904-0495, Japan
4 Centre for Life's Origins and Evolution, Department of Genetics, Evolution and Environment, University College London, London WC1E 6BT, United Kingdom
5 Radcliffe Department of Medicine, Weatherall Institute of Molecular Medicine, University of Oxford, Oxford OX3 9DS, UK
6 School of Fishery, Zhejiang Ocean University, Zhoushan, 316022, China
7 Laboratory for Marine Biology and Biotechnology, Qingdao National Laboratory for Marine Science and Technology, Qingdao, China

*Author for correspondence: xufei@qdio.ac.cn

Supplementary Table 1:

Comparison of amino acid sequence between the deduced A-chains and B-chains of human insulin/IGF, *Drosophila* ILPs and mollusc ILPs.

	B-chain	A-chain
**Human**		
INS	FVNQHLCGSHLVEALYLV-CGERGFFYTPKT	GIVEQCCTSI-CSLYQLENYCN*
IGF1	GPETLCGAELVDALQFV-CGDRGFYFNKPT	GIVDECCFRS-CDLRRLEMYCA
IGF2	AYRPSETLCGGELVDTLQFV-CGDRGFYFSRPA	GIVEECCFRS-CDLALLETYCA
**Fly**		
DILP1	MVTPTGSGHQLLPPGNHKLCGPALSDAMDVV-CPHGFNTLP	HLTGGVYDECCVKT-CSYLELAIYCLPK*
DILP2	LCSEKLNEVLSMV-CEEYNPVIPH	TRQRQGIVERCCKKS-CDMKALREYCSVVRN*
DILP3	TMKLCGRKLPETLSKL-CVYGFNAMT	LRDGVFDECCLKS-CTMDEVLRYCAAKPRT*
DILP4	LQPVQGRRKMCGEALIQALDVI-CVNGFT	IAHECCKEG-CTYDDILDYCA*
DILP5	ANSLRACGPALMDMLRVA-CPNGFNSMFA	DFRGVVDSCCRKS-CSFSTLRAYCDS*
DILP6	SPLAPTEYEQRRMMCSTGLSDVIQKI-CVSGTVA	DLQNVSDLCCKSGGCTYRELLQYCKG*
DILP7	LQHTEEGLEML**F**RERSQSDWENV**WH**QETHSRCRDK**L**VRQLYW**A**-CEK**D**I**Y**RLT	SDGNTPSISN**E**CCTKAGCT**WEE**Y**AE**YCPSNKRRNHY*
DILP8	SFCSLERMKKFAMEACEHLFQADEGA	DHSSRSYNNIPYCCLNQ-CEEEFF---C*
**Oyster**		
MIP123	GFEKVCTFETYRRGVHQQGACGDNLADMLRLV-CRKY	YGDINIVCECCYHS-CSVAEFEDYCAE*
MIP4	DFERVCNSQTDLRGPDPQGICGRLIPEMLHLV-CGGQYYVPS	NAYQGIVCECCYHG-CNWFELQQYCGF
MILP7	AAIHYQSYNPV**F**LQRTEAEWRSL**WH**NDCHRVCHFE**L**DQHVDL**A**-CRM**D**I**Y**RIR	NVLN**E**CCYSKGCS**WEE**F**AE**FCQFSIRLPATNANSCVS*
ILP	SQLQACGSALTDILSLV-CRNQFHAPA	GGVVEECCFSS-CSYENLLLYCS
**Lymnaea**		
MIP1	QFSACNINDRPHRRGVCGSALADLVDFA-CSSSNQPAMV	QGTTNIVCECCMKP-CTLSELRQYCP*
MIP2	QSSCSLSSRPHPRGICGSNLAGFRAFI-CSNQNSPSMVKRDAETGWLLPETMV	QRTTNLVCECCFNY-CTPDVVRKYCY*
MIP3	TTQHTCSILSRPHPRGLCGSTLANMVQWL-CSTYTTSSKV	ESRPSIVCECCFNQ-CTVQELLAYC*
MIP5	QFSACSFSSRPHPRGICGSDLADLRAFI-CSRRNQPAMVKRDAETGWLLPETMV	QRTTNLVCECCYNV-CTVDVFYEYCY*
MIP7	QQVNTCTMFSRQHPRGLCGNRLARAHANL-CFLLRNTYPDIFPR	EVMAEPSLVCDCCYNE-CSVRKLATYC*
**Aplysia**		
AILP1	NFEHSCNGYMRPHPRGLCGEDLHVIISNL-CSSLGGNRRFLAKYMV	EASGSITCECCFNQ-CRIFELAQYCRLPDHFFSRIS
AILP2	ETRYCRPGFSRPHPRGFCGSALARLHANF-CLLLRWAYPEHFPMG	AARGKRSLVCDCCYSA-CDERKLAVY?*
AILP3	QLVCVRDSRPHPRGICGSRLTRAHNNL-CFLLSRTYPEHFPG	GGTQSNMVCDCCYHM-CSPRQLATYC*
AILP4	ERRLCGQLLADTLDMV-CEDRGFNLA	EVNSRIVDECCLRP-CNFATLQSYCADPDDPVVEIPEDVL

Note: Conserved cysteine residues shaded and bold. Additional cysteine residues underlined and bold. * indicates C-terminus of protein. ? indicates a putative "C" inferred from the structure character of insulin superfamily. Bold letters in the A&B chains of DILP7 and ILP3 shows the identical amino acids they share (FWWHHNEGWEEAE) that distinguish them from other members of insulin superfamily.

Supplementary Table 2:

Reads Per Kilobase per Million mapped reads (RPKM) of oyster *ILPs* and *Pdx* in main oyster tissues calculated by RNAseq.

gene	mantle	digestive gland	gill	adductor muscle	hemolymph	labial palps	male gonad	female gonad
cgILP1	0.00	24.16	0.66	0.00	0.00	1.51	12.62	1.83
cgILP2	2.47	14.94	0.36	0.00	0.00	0.38	11.48	3.65
cgILP3	0.39	0.11	0.00	0.20	0.00	0.08	0.13	0.25
cgILP4	2.03	31.52	0.44	0.00	0.00	0.00	24.97	2.09
cgPDX	0.31	18.62	54.99	0.71	0.97	0.08	10.02	61.67

Note: data were calculated according to raw data of RNAseq from Zhang et al. 2012.

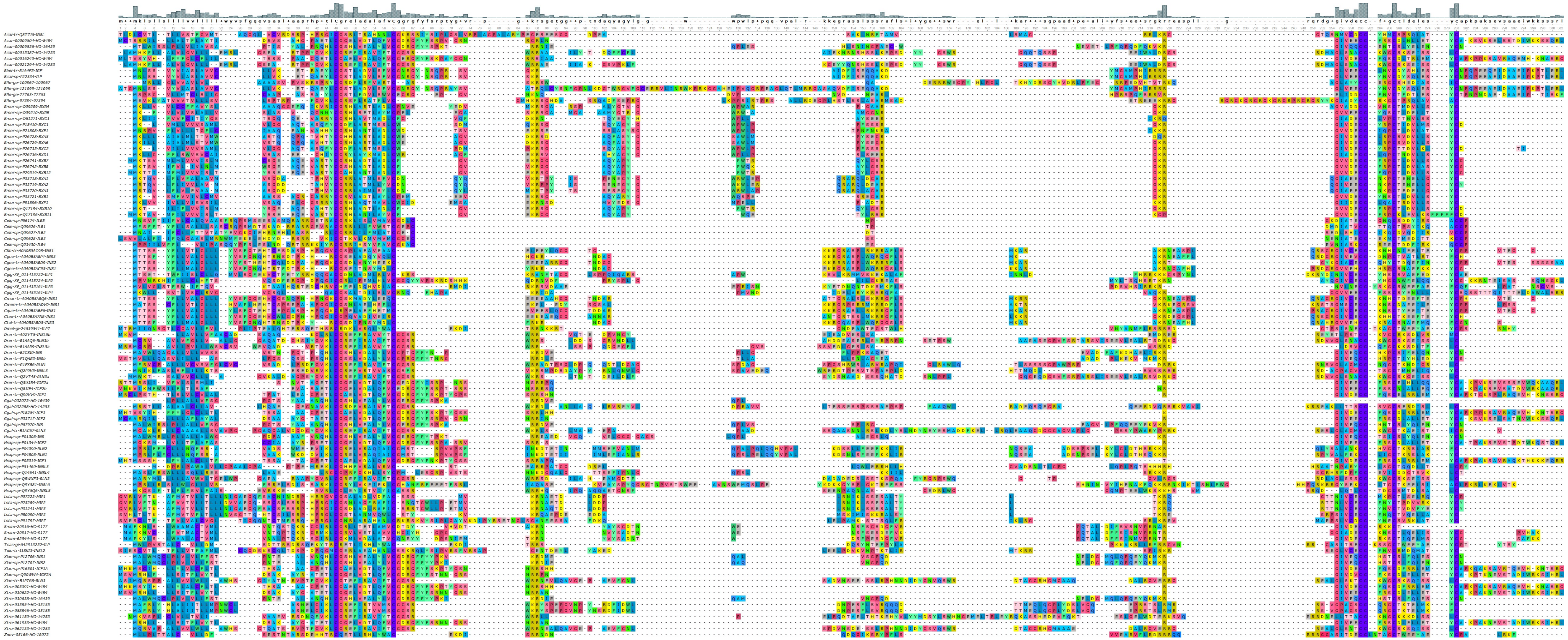

Supplementary Fig. 1

Alignment of 101 peptide sequences in the insulin superfamily. Species abbreviations are given in Supplementary Data 1.

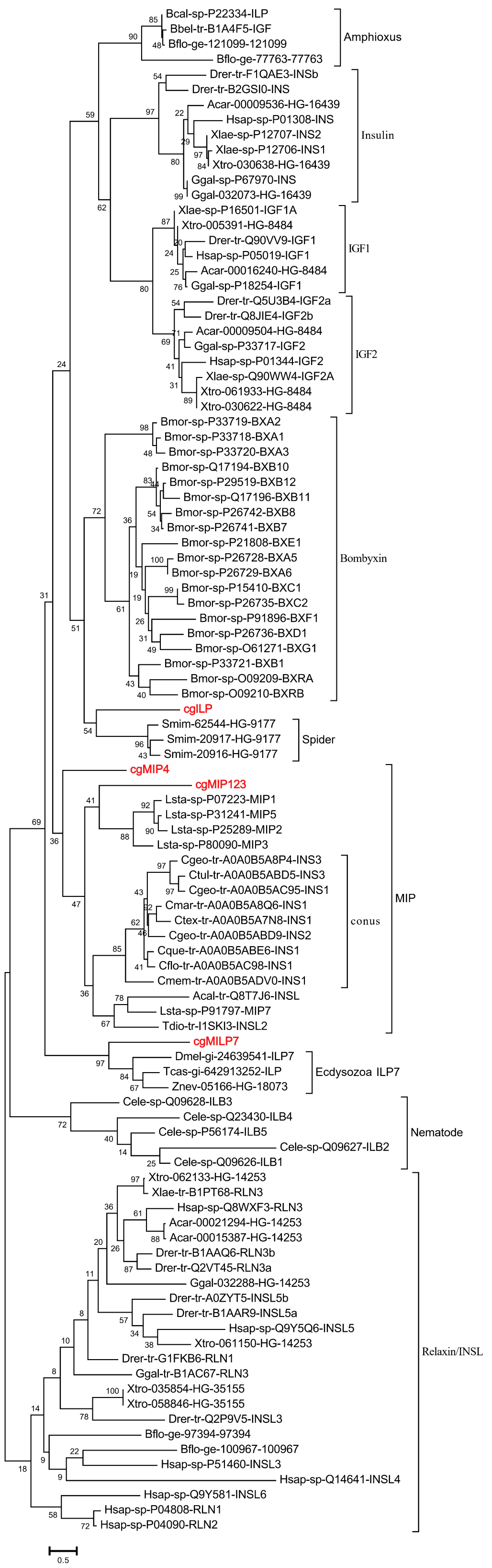

Supplementary Fig. 2

The full phylogenetic tree (RAxML method) of insulin-related genes used in the main text Figure 1.

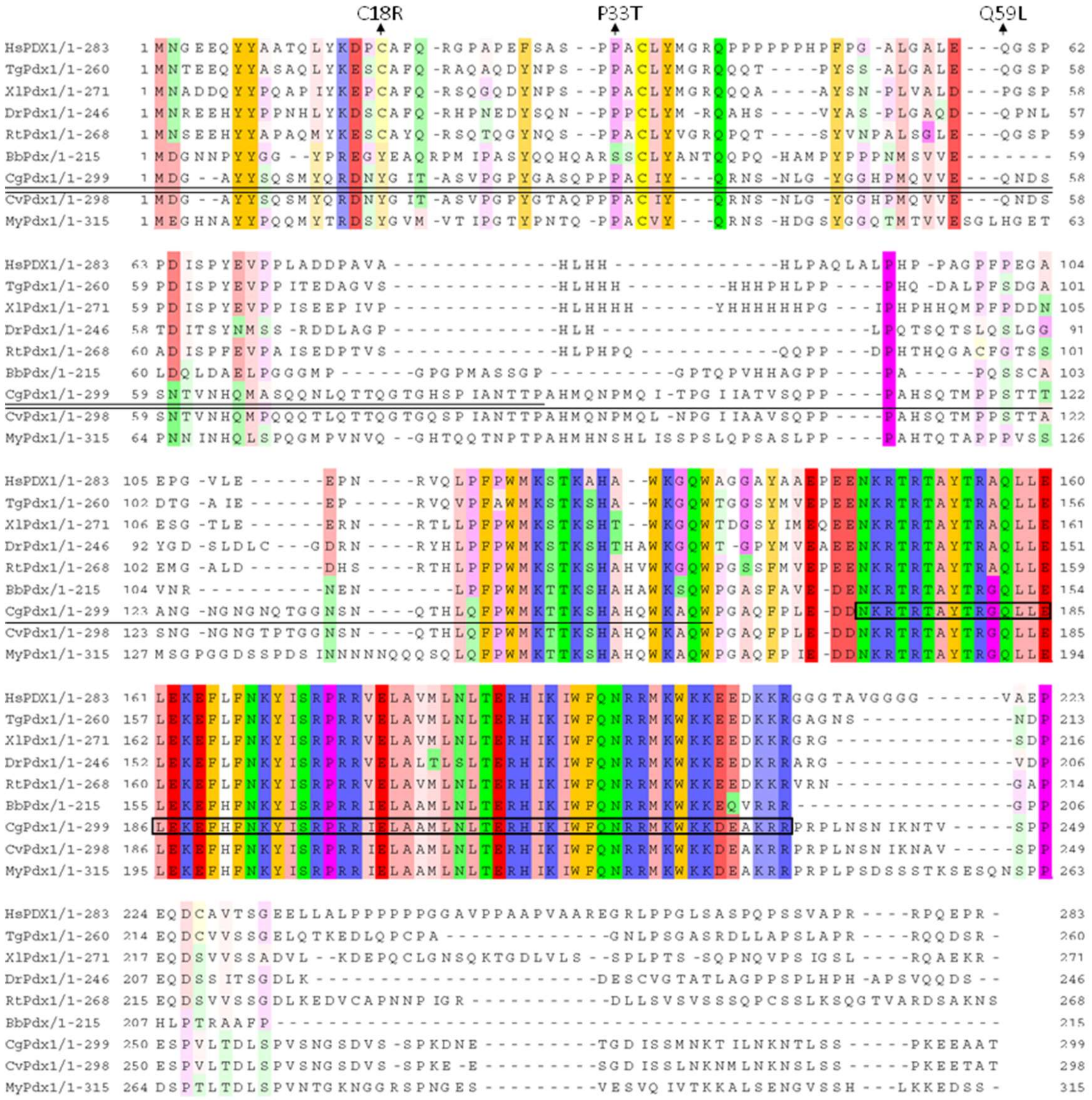

Supplementary Fig. 3

Alignment of Pdx proteins. Hs, *Homo sapiens*; Tg, *Taeniopygia guttata*; Xl, *Xenopus laevis*; Rt, *Rhincodon typus*; Bb, *Branchiostoma belcheri*; Dr, *Danio rerio*; Cg, *Crassostrea gigas*; Cv, *Crassostrea virginica*; My, *Mizuhopecten yessoensis*. Boxed sequence is the homeodomain of oyster *C. gigas* Pdx. Double-underlined sequence is the predicted homology transactivation domain (TAD), while peptide marked with both double and single underlines is designed as antigen to produce antibodies E9112 and E9113. Marked sites were mutations known to be responsible for the development dabetes: C18R and Q59L are found in patients with Type 2 Diabetes, while P33T contributes to the development of maturity-onset diabetes of young 4.

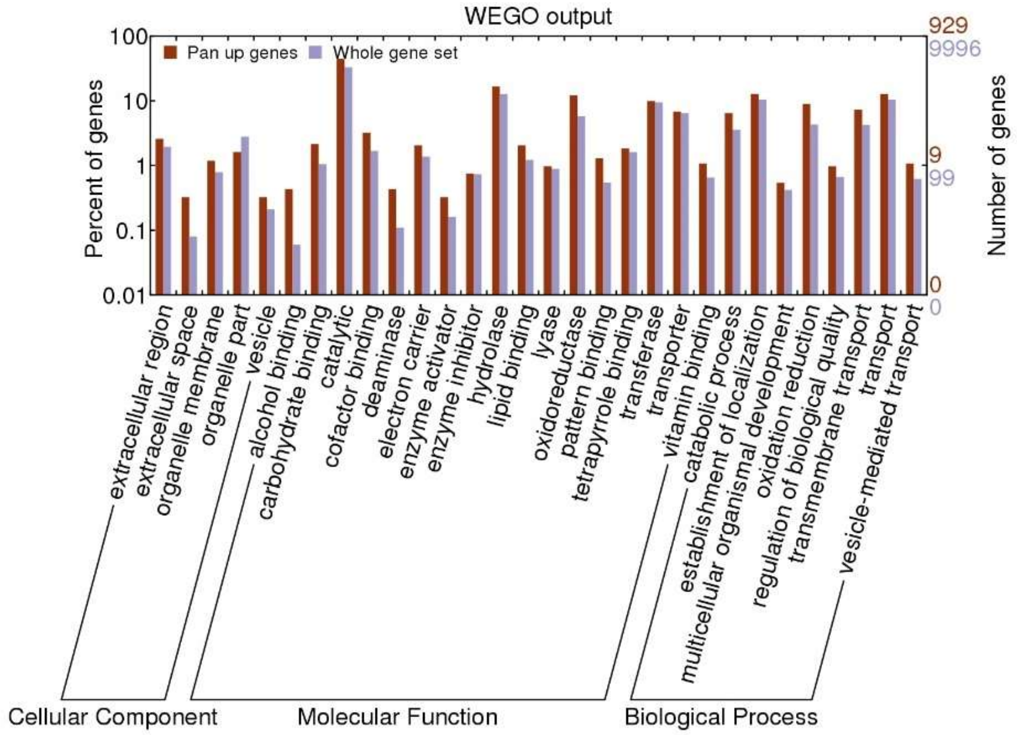

Supplementary Fig. 4

GO terms enrichment of genes enriched in oyster hepatopancreas compared to other tissues (mantle, gill, labial palps, heart, hemolymph, adductor muscle, male gonad, female gonad).

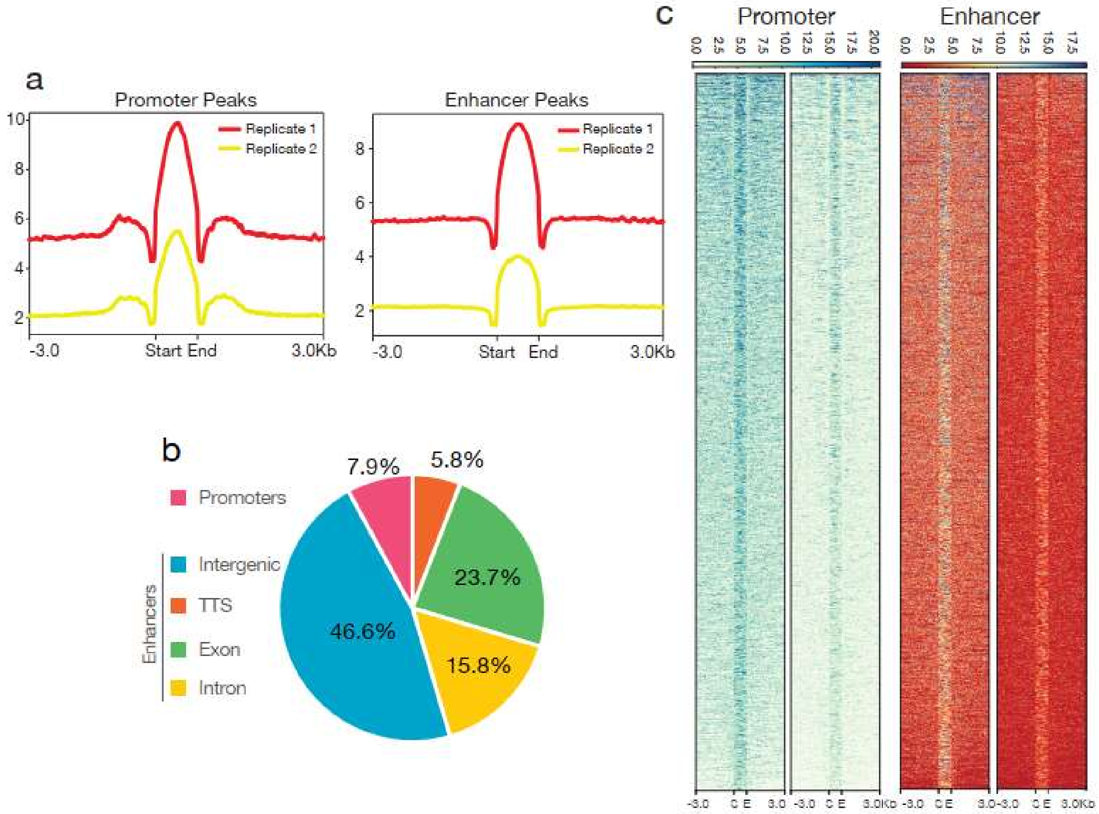

Supplementary Fig. 5

Assessment of the quality of oyster hepatopancreas ATAC-seq data. **a** Distribution of open chromatin peaks relative to closest gene. **b&c** Profile plot and heatmaps of putative promoter (green heatmap) and enhancer peaks (red heatmap) in two ATAC-seq replicates.

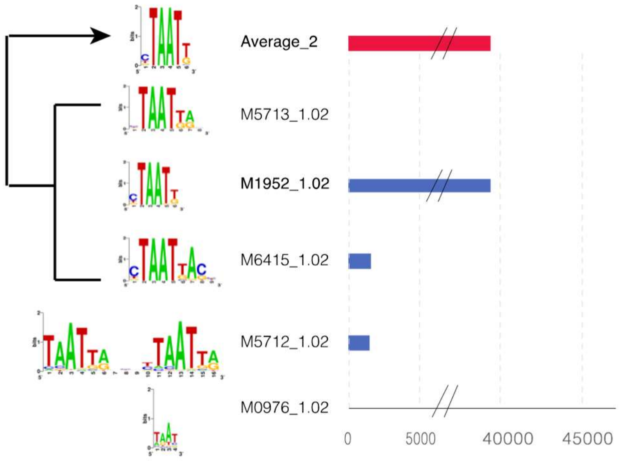

Supplementary Fig. 6

Details of Pdx consensus PWM generation and relative number of instances of each motif. This figure shows the Logos of position weighted matrices (PWMs) utilized to generate consensus Pdx PWM Average_2 and barplot indicating number of instances each motif was detected in open chromatin regions.

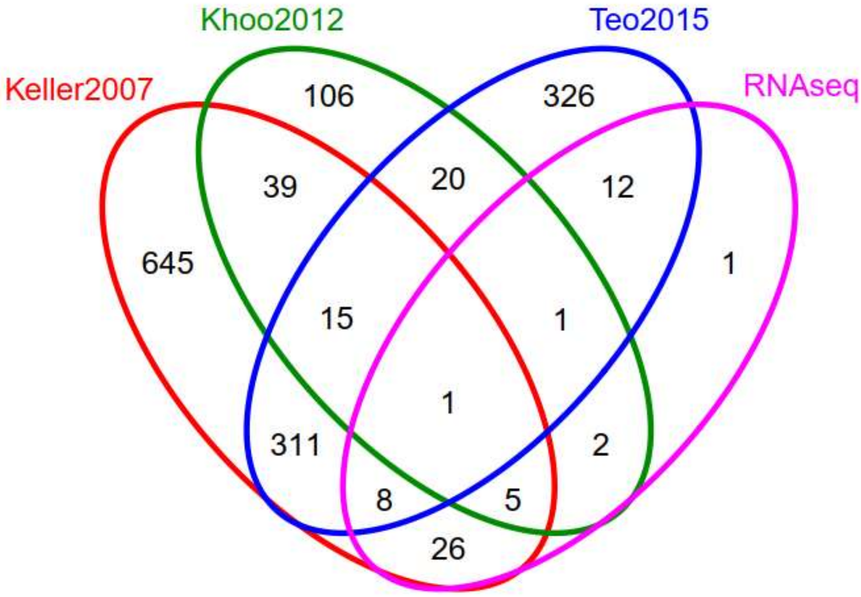

Supplementary Fig. 7

Distribution of oyster orthologs identified to contain Pdx regulation motif in human or mouse. Dataset “Keller2007” was from the Pdx motif containing orthologs identified in mouse by Keller et al. 2007. Dataset “Khoo2012” was from the Pdx motif containing orthologs identified in human cells by Khoo et al. 2012. Dataset “Teo2015” was from the Pdx motif containing orthologs identified in human cells by Teo et al. 2015. Dataset “RNAseq” was the oyster hepatopancreas enriched genes identified by this study. Source data are provided in Supplementary Data 3.

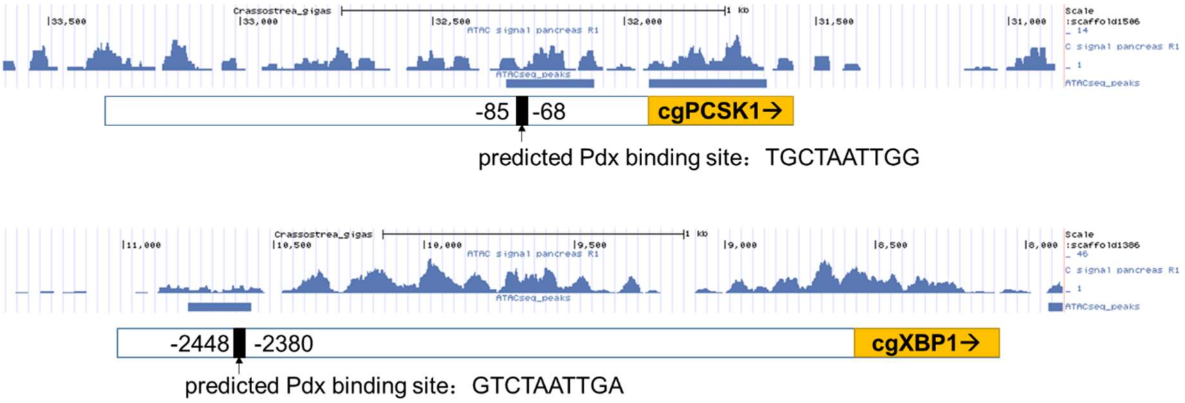

Supplementary Fig. 8

Distribution of potential Pdx binding site upstream *cgPCSK1* and *cgXBP1*. The ATAC-seq peaks are not direct extrapolation of the regions with the highest sequencing signal, as the calling process takes into account both replicates, the averaged background over a window, as well as the shift induced by the library construction process. Note that the assay on *cgXBP1* was limited by the poor genome assembly where three copies were predicted (LOC105332313 in scaffold663, and LOC105344684 & LOC105344694 in scaffold1386).

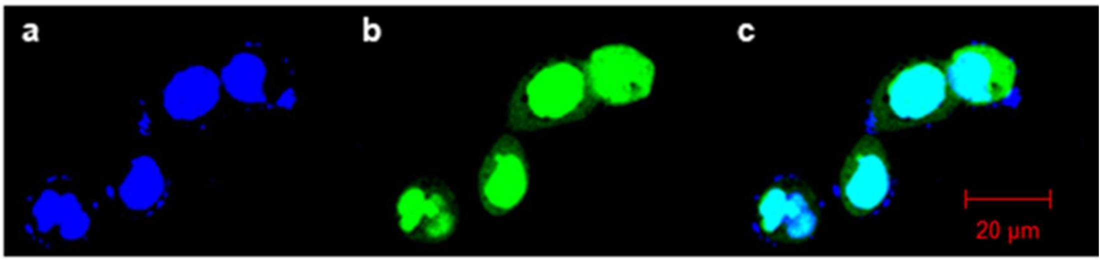

Supplementary Fig. 9

Nuclear localization of oyster Pdx protein in HeLa cells. **a** Nuclei stained by Hoechst (Blue). **b** Oyster Pdx-green fluorescent protein (GFP) fusion (green). **c** Merged image. Observations from single experiment.

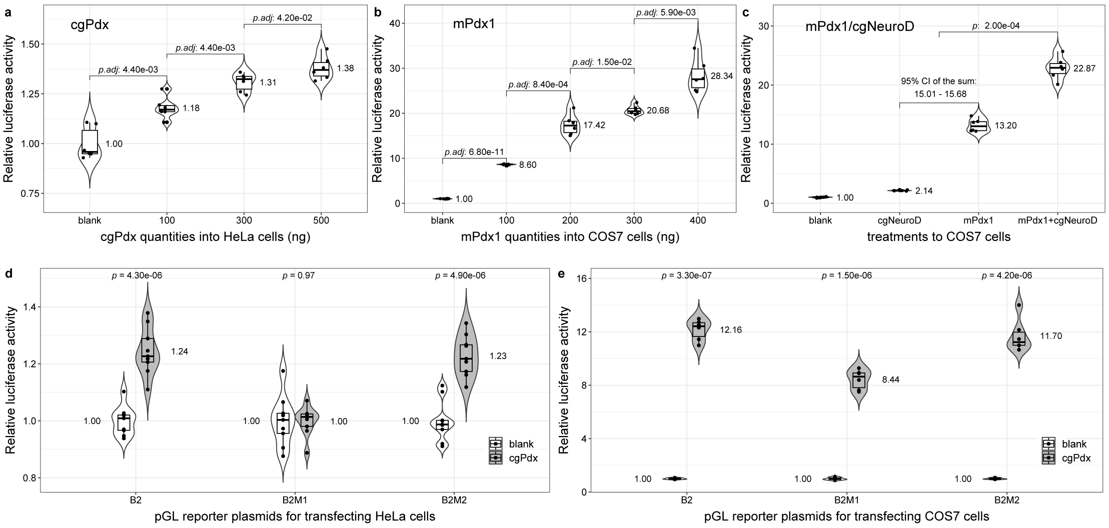

Supplementary Fig. 10

functional characterization of cgPdx, mPdx1 and cgNeuroD proteins in HeLa and COS7 cells. **a** Gradient experiment of oyster cgPdx in HeLa cells demonstrating that responsiveness of the B1 cis-element is dependent on the quantity of the transfected cgPdx expression plasmid. **b** Gradient experiment of mouse mPdx1 in COS7 cells showing dose dependent and stronger induction to the reporter gene transcription. **c** mPdx1-cgNeuroD co-transfection gives higher transcriptional activity than the sum of the individual activities, showing the synergy between mPdx1 and cgNeuroD. 95% confidence interval (CI) of the sum between cgNeuroD and mPdx1 was estimated based on all the possible combinations between the relative luciferase values from cgNeuroD and mPdx1. Significance was calculated by bootstrapping test (*n*=100000), in which sum values with the same sample size with the group (mPdx1+cgNeuroD) was randomly sampled from all the possible combinations between group cgNeuroD and group mPdx1. **d** Mutating the TTCTAATTAC site (B2M1) destroys the ability of oyster cgPdx to activate luciferase expression in HeLa cells, while mutation of a different TAAT site (B2M2) does not. Relative luciferase activity in the cells transfected with blank pSI and the same reporter plasmid is set at 1.0. **e** In COS7 cells, mutant B2M1 decrease the activation level of reporter gene, while mutant B2M2 shows no influence on the transcription activity. For all experiments, *n*=3 biologically independent cells by 2 technically independent measurements. *p* values were calculated by two tailed Student's t test and Holm's method to correct for multiple comparisons (*p.adj*). Source data are provided as a Source Data file.

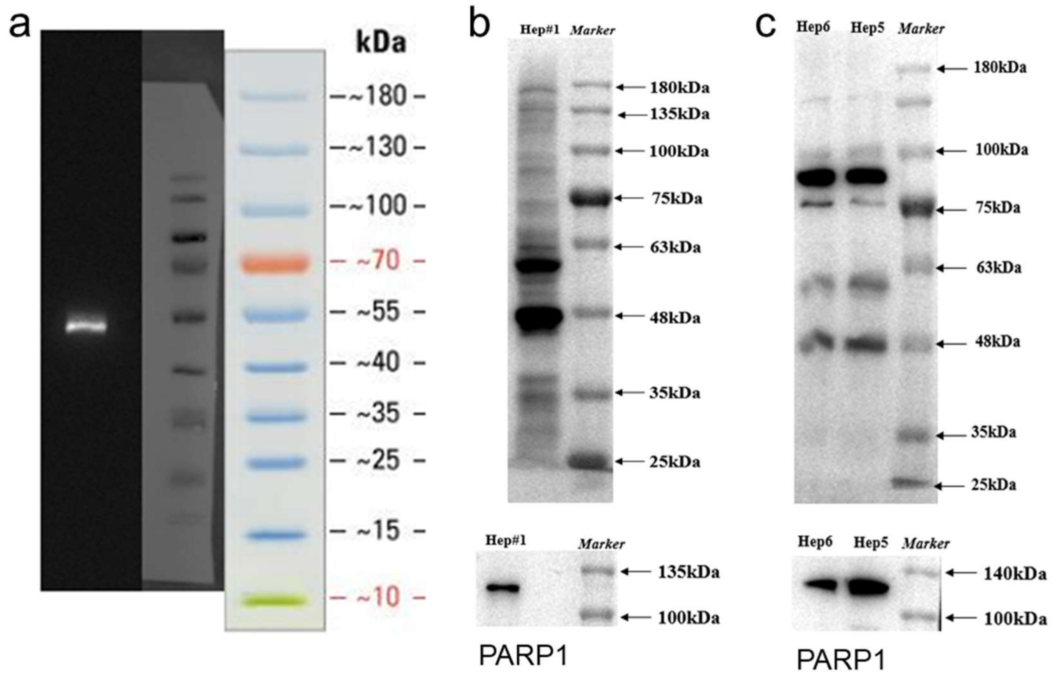

Supplementary Fig. 11

Western blot assay on cgPdx expressed in HEK293T cells (a) and for validation of antibodies E9112 (b) and E9113 (c). cgPdx (with V5 tag) expressed in HEK293T cells showed ~50 KDa molecular weight (anti-V5 antibody was applied). Oyster hepatopancreas (Hep#1, Hep5 and Hep6) was used to validate antibodies E9112 (b) and E9113 (c). A band was observed around the expected molecular weight of cgPdx. Anti-PARP1 antibody (ab32138, abcam, Shanghai, China) was used as positive control. Blots were developed with peroxidase-conjugated goat anti-rabbit IgG (H+L) secondary antibodies (33101ES60, Yeasen, Shanghai, China) at 1/10000 dilution for 1 hour at room temperature before imaging. Dilutions was 1/1000 for E9112 and E9113, 1/5000 for ab32138. Observations from single experiment. Source data are provided as a Source Data file.
