# Supplementary material for: Evidence from oyster suggests an ancient role for Pdx in regulating insulin gene expression in animals
Source: Nat Commun. 2021 May 25;12:3117. doi: 10.1038/s41467-021-23216-7 (PMC8149454; doi:10.1038/s41467-021-23216-7)
Supplement: Supplementary file 2 — Description of Additional Supplementary Files [file 41467_2021_23216_MOESM2_ESM.docx]

**Description of Additional Supplementary Files**

File Name: Supplementary Data 1

Description: Species and sequence accessions used for phylogenetic analysis of insulin-related peptides.

File Name: Supplementary Data 2

Description: Oyster hepatopancreas-enriched genes.

File Name: Supplementary Data 3

Description: Oyster orthologues identified to contain putative Pdx regulation motif in human or mouse.

File Name: Supplementary Data 4

Description: Primers used in this study.
