# Supplementary material for: Evidence from oyster suggests an ancient role for Pdx in regulating insulin gene expression in animals
Source: Nat Commun. 2021 May 25;12:3117. doi: 10.1038/s41467-021-23216-7 (PMC8149454; doi:10.1038/s41467-021-23216-7)
Supplement: Supplementary file 8 — Reporting Summary [file 41467_2021_23216_MOESM8_ESM.pdf]

nature research

Corresponding author(s): Fei Xu

Last updated by author(s): Jan 20, 2021

Reporting Summary

Nature Research wishes to improve the reproducibility of the work that we publish. This form provides structure for consistency and transparency in reporting. For further information on Nature Research policies, see our Editorial Policies and the Editorial Policy Checklist.

Statistics

For all statistical analyses, confirm that the following items are present in the figure legend, table legend, main text, or Methods section.

n/a	Confirmed	
☐	☒	The exact sample size (*n*) for each experimental group/condition, given as a discrete number and unit of measurement
☐	☒	A statement on whether measurements were taken from distinct samples or whether the same sample was measured repeatedly
☐	☒	The statistical test(s) used AND whether they are one- or two-sided<br>*Only common tests should be described solely by name; describe more complex techniques in the Methods section.*
☐	☒	A description of all covariates tested
☐	☒	A description of any assumptions or corrections, such as tests of normality and adjustment for multiple comparisons
☐	☒	A full description of the statistical parameters including central tendency (e.g. means) or other basic estimates (e.g. regression coefficient) AND variation (e.g. standard deviation) or associated estimates of uncertainty (e.g. confidence intervals)
☐	☒	For null hypothesis testing, the test statistic (e.g. *F*, *t*, *r*) with confidence intervals, effect sizes, degrees of freedom and *P* value noted<br>*Give P values as exact values whenever suitable.*
☒	☐	For Bayesian analysis, information on the choice of priors and Markov chain Monte Carlo settings
☐	☒	For hierarchical and complex designs, identification of the appropriate level for tests and full reporting of outcomes
☒	☐	Estimates of effect sizes (e.g. Cohen's *d*, Pearson's *r*), indicating how they were calculated

Our web collection on statistics for biologists contains articles on many of the points above.

Software and code

Policy information about availability of computer code

Data collection	No software was used for data collection
Data analysis	softwares: MAFFT (v. 7.221), TrimAl (v1.4), RAxML (v. 8.2.12), UGENE (v. 1.28.0), SignalP (v. 4.1), Bowtie (v. 2.1.0), RepeatModeler (v. 1.0.11), MACS2 (v. 2.1.1), Homer (v.4.9), Gimme motifs suite (v.0.11.1), HISAT2 (v. 2.1.0), StringTie (v. 1.3.4), Ballgown (2.0.0), edgeR (3.14.0), R (4.0.3) algorithms: DUST and TRF algorithms for repeat elements prediction.

For manuscripts utilizing custom algorithms or software that are central to the research but not yet described in published literature, software must be made available to editors and reviewers. We strongly encourage code deposition in a community repository (e.g. GitHub). See the Nature Research guidelines for submitting code & software for further information.

Data

Policy information about availability of data

All manuscripts must include a data availability statement. This statement should provide the following information, where applicable:

- Accession codes, unique identifiers, or web links for publicly available datasets
- A list of figures that have associated raw data
- A description of any restrictions on data availability

Accession codes: The raw sequencing data and analysis results for C. gigas hepatopancreas RNAseq and ATAC-seq have been deposited in the Gene Expression Omnibus with accession number GSE107713 under BioProject PRJNA417263. Mapping details of reads onto the genome and ATAC-seq peak calling result can also be visualised by adding the hub http://zoo-animalia.zoo.ox.ac.uk/Cragig/hub2.txt into UCSC genome browser. The authors declare that the main data supporting the findings of this study are available within the article and its Supplementary Information or from the authors upon reasonable request. The source data underlying Figures 2a, 5a–f, Supplementary Fig. 10a-e and 11a-c are provided as a Source Data file.

Field-specific reporting

Please select the one below that is the best fit for your research. If you are not sure, read the appropriate sections before making your selection.

☒ Life sciences ☐ Behavioural & social sciences ☐ Ecological, evolutionary & environmental sciences

For a reference copy of the document with all sections, see nature.com/documents/nr-reporting-summary-flat.pdf

Life sciences study design

All studies must disclose on these points even when the disclosure is negative.

Sample size	RNAs from multi individuals were usually mixed as one sample to minimize individual variations during the gene expression pattern study of marine invertebrates. Here we chose a sample size of three considering both reliability and operability, which is also a commonly considered number in other similar studies.
Data exclusions	No exclusion was performed to the data in this study.
Replication	1. Realtime qPCR: To minimize variations of gene expression levels, three biological and three technical replicates were usually recommended. Here we performed realtime qPCR with one biological and three technical replicates, in which RNAs from three individuals were equally mixed as one sample for each tissue to minimize individual variations. As all the target genes (cgILPs and cgPdx) demonstrated obvious tissue-specific expression pattern reported by previous RNAseq or realtime qPCR studies, one biological (one sample with mixed RNA from three individual) and three technical replicates were conducted to simply verify previous observations, and the result is fine to support the conclusion.<br>2. in situ hybridization (ISH): when negative control was conducted well, few replications were conducted for ISH on sections, especially when the signal is clear and corresponding to the result of other assays (e.g. realtime qPCR). In this study, we observed clear signal from ISH and consistent results with qPCR.<br>3. ATACseq: As different replicates would only be used to verifying each other, and no statistical calculations would be performed based on the replicates, we designed two biological replications for ATACseq experiment. The two replicates showed high reproducibility ($r^2 > 0.88$) as has been shown in the result section of the paper.<br>4. RNAseq: To control the false discovery rate, as many replicates as possible was usually suggested during differentially expressed gene identification. Some report recommended at least six biological replicates (Schurch et al. 2016). In this study, we analyzed oyster tissue enriched genes mainly relied on public RNAseq data, which were only with one replicate. To verify hepatopancreas enriched genes, we further sequenced RNAs extracted from oyster hepatopancreas tissue. We used these data only aimed to make a general conclusion on the function of oyster hepatopancreas, which is not the main focus of this study.<br>5. Dual luciferase assay: Three biological replicates were usually recommended for dual luciferase assay, we thus set three biological replicates with two technical replicates for each assay. All showed consistent results.<br>6. ChIP-qPCR: There was a "two-replicate guideline" for ChIP experiment (Rozowsky et al. 2009), especially for that with a binary determination (binding or not), which is applicable for this study. We indeed observed reproducible results within the two replicates. Furthermore, we observed similar ChIP results with another independent antibody which added confidence.
Randomization	For Realtime qPCR, in situ hybridization, ATACseq, RNAseq, and ChIP-qPCR, the sampling of oyster tissues was randomly conducted, dissection site and tissue pieces were all picked randomly. Cell seeding and transfection were all conducted by randomized design during dual luciferase assay experiment.
Blinding	As it is necessary to avoid the bias from subjectivity of the experiment target of researcher, blinding design is usually applied in studies on human. In this study, all the experiment objects were no life, thus no investigators need to be blinded. During testing, almost all experimental groups were treated with machine. Only in situ hybridization were determined by naked eye. We did not distinguish sense or antisense probes during all the ISH experiment to keep all groups were treated with the same conditon. The probe information was only considered during microscopy observation and photo analysis.

Reporting for specific materials, systems and methods

We require information from authors about some types of materials, experimental systems and methods used in many studies. Here, indicate whether each material, system or method listed is relevant to your study. If you are not sure if a list item applies to your research, read the appropriate section before selecting a response.

Materials & experimental systems

n/a	Involved in the study	
☐	☒	Antibodies
☐	☒	Eukaryotic cell lines
☒	☐	Palaeontology and archaeology
☐	☒	Animals and other organisms
☒	☐	Human research participants
☒	☐	Clinical data
☒	☐	Dual use research of concern

Methods

n/a	Involved in the study	
☒	☐	ChIP-seq
☒	☐	Flow cytometry
☒	☐	MRI-based neuroimaging

Antibodies

Antibodies used	Rabbit anti-cgPdx polyclonal antibodies, E9112 and E9113, were developed by Abclonal (Wuhan, China). Monoclonal anti-PARP1 (ab32138) from clone number E102 was purchased from abcam (Shanghai, China). Polyclonal peroxidase-conjugated goat anti-rabbit IgG (H+L) secondary antibodies was purchased from Yeasen (Shanghai, China) whose source of the antibody was reported from Jackson Immuno Research Laboratories (Code number: 111-035-003).
Validation	1. Oyster cgPdx antibodies: Western blot assay was conducted on cgPdx expressed in HEK293T cells to determine the molecular weight. Validation of antibodies E9112 and E9113 was performed with tissue lysate of oyster hepatopancreas. cgPdx (with V5 tag) expressed in HEK293T cells showed ~50 kDa molecular weight (anti-V5 antibody was applied). Western blotting on oyster hepatopancreas produced a band of the expected molecular weight for cgPdx. Observed band size for E9112: 50, 58 kDa, for E9113: 50, 58, 80, 90 kDa.<br>2. Anti-PARP1 (ab32138): A rabbit recombinant monoclonal [E102] to PARP1 recognizing both pro-form and p25 cleaved form of PARP1. This antibody reacts with human and was predicted to work with mouse and rat. The predicted band size is 113 kDa. Manufacturer's website listed three western blot notes, with observed band size at 125 kDa (in HAP1 and HeLa cells), 113 kDa (in HEK-293T cells), and (120, 25) kDa (in Jurkat cells) respectively. There were also three customer reviews with observed band size at (113, 85) kDa (RPE choroid tissue), (113, 60) kDa (THP1 Cells), and a band between 100-140 kDa (mouse Kidney). When applying this antibody to oyster hepatopancreas, two types of bands were observed: one with a single band between 100-135 kDa, the other with (100-140, 25, 40) kDa as well as several weak bands. This may reflect that different cell types/status were sampled during the two experiments, because of the complex tissue composition of oyster hepatopancreas.

Eukaryotic cell lines

Policy information about cell lines

Cell line source(s)	Both HeLa and COS-7 were originally from ATCC, USA, while the later one was kindly provided by Dr Qingfeng Yan, Zhejiang University, Zhejiang, China.
Authentication	None of the cell lines used were authenticated.
Mycoplasma contamination	The cell lines were not tested for mycoplasma contamination.
Commonly misidentified lines<br>(See ICLAC register)	No misidentified lines was used in this study.

Animals and other organisms

Policy information about studies involving animals; ARRIVE guidelines recommended for reporting animal research

Laboratory animals	The study did not involve laboratory animals.
Wild animals	Three-year old Pacific oysters (Crassostrea gigas) used in this study were from the Oxford Covered Market, reportedly collected from the Scottish coast. As oyster shells were tightly closed, all animals were opened with oyster knife and killed for qPCR and in situ hybridization, RNA-seq, ATAC-seq and gene cloning studies.
Field-collected samples	Collected oysters were acclimated in artificial seawater at 16°C for 7 days before use.
Ethics oversight	As oysters are invertebrate and samples were collected from market sold as food, no ethical approval or guidance required.

Note that full information on the approval of the study protocol must also be provided in the manuscript.
